# Fuzzy Logic Approach for Modeling of Heating and Scale Formation in Industrial Furnaces

**DOI:** 10.3390/ma17215355

**Published:** 2024-11-01

**Authors:** Jaroslaw Krzywanski, Jaroslaw Boryca, Dariusz Urbaniak, Henryk Otwinowski, Tomasz Wylecial, Marcin Sosnowski

**Affiliations:** 1 Department of Advanced Computational Methods, Faculty of Science and Technology, Jan Dlugosz University in Czestochowa, 13/15 Armii Krajowej Ave., 42-200 Czestochowa, Poland; j.krzywanski@ujd.edu.pl (J.K.); m.sosnowski@ujd.edu.pl (M.S.); 2 Faculty of Production Engineering and Materials Technology, Czestochowa University of Technology, 19 Armii Krajowej Ave., 42-201 Czestochowa, Poland; jaroslaw.boryca@pcz.pl (J.B.); tomasz.wylecial@pcz.pl (T.W.); 3 Faculty of Mechanical Engineering, Czestochowa University of Technology, 21 Armii Krajowej Ave., 42-201 Czestochowa, Poland; dariusz.urbaniak@pcz.pl

**Keywords:** fuzzy logic, artificial intelligence, simulation, heating, scale, steel charge, loss of steel, energy saving

## Abstract

Heating steel charges is essential for proper charge formation. At the same time, it is a highly energy-intensive process. Limiting the scale formed is critical for reducing heat consumption in this process. This paper applies fuzzy logic to model heating and scale formation in industrial re-heating furnaces. Scale formation depends on the temperature of the initial charge, heating time, excess air coefficient value, and initial scale thickness. These parameters were determined based on experimental tests, which are also the inputs in the model of the analyzed process. The research was carried out in walking beam furnaces operating in hot rolling mill departments. To minimize the excess energy consumption for heating a steel charge in an industrial furnace before forming, a heating and scale formation (HSF) model was developed using the fuzzy logic-based approach. The developed model allows for the prediction of the outputs, i.e., the charge’s final surface temperature and the scale layer’s final thickness. The comparison between the measured and calculated results shows that the model’s accuracy is acceptable.

## 1. Introduction

The global energy crisis, triggered by a series of global factors, has necessitated the search for new conditions under which technological processes can be conducted [1,2]. The ongoing war in Ukraine has reshaped the global fuel structure. The supply routes of various energy fuels have changed, and their prices have increased. Additionally, there is a growing trend toward a total transformation of the existing energy and environmental order toward a zero-emission lifestyle, especially in Europe [1,2]. Regulations are being introduced that mandate fossil fuels be abandoned in favor of renewable energy sources. Previously, regulations were primarily focused on the “large-scale energy sector”, but now regulations aimed at changes at the individual consumer level, such as the Buildings Directive [1] or the phasing out of internal combustion vehicles [2], are becoming increasingly prevalent. In this context, producing many goods under the existing technological regimes has become unprofitable. Technologies requiring gas or electricity have become either less competitive or unprofitable. This has increased the need for empirical research and model studies to establish new conditions for optimizing technological processes [3,4,5,6,7,8,9,10]. One such process is heating steel charges before further processing in steel mills.

Modern industrial-scale reheating furnaces used in metal and metal alloy forming are complex thermal devices [11]. Their operation is based on intricate heat and mass transfer phenomena, and proper operation requires control of the primary process parameters during the heating of the charge before further forming. The amount of mill scale formed can be determined by the following quantities: the mass of scale relative to the unit surface area or mass of the charge, the steel loss per unit mass of scale, and the thickness of the scale layer [12,13]. A critical issue due to the intensity of the heating process is the impact of the scale layer thickness on the heat transfer conditions inside the charge [12,13]. Scale, compared to steel, has a significantly lower thermal conductivity coefficient, which negatively affects the heating intensity, leading to increased heat consumption or inducing uneven temperature distribution in the cross-section of the charge [12,13]. Steel loss is a quantity that can be determined experimentally. However, the methodology for its measurement is challenging owing to the high temperature of the charge and the need to interfere with the technological process [14]. Therefore, heating a steel charge in industrial furnaces presents extremely complex challenges when creating a determined mathematical model. Demin et al. [15] present a novel method to solve the heat transfer boundary condition of billet in a reheating furnace by combining a black box test with the mathematical model. The heating of a charge in the form of a bundle of round steel rods was studied in [16]. The heat flow through the steel bars was described using a mathematical model that considered the formation of an oxidized iron layer in the form of scale on the surface of the bars. The model is based on the analysis of thermal resistances present in the examined charge, which is treated as a porous medium with a granular structure [16].

Comprehensive heat treatment removes defects in castings manufactured by the aluminothermic method [17]. Annealing is employed when chemical and structural inhomogeneities, as well as high internal stresses, are present. Heat treatment enables the properties of the cast semi-finished products to be raised to the required level [17].

Paper [18] presents the results of a study of the heating rate in a rotary furnace of a long charge made of three different materials about changes in the atmosphere inside the furnace. Numerical calculations were conducted based on a developed heat transfer model in the rotary furnace chamber, considering the increase in the thickness of the scale layer. Frydrych et al. [19] discussed the application of machine learning in steelmaking processes. Their article presents two issues: one related to classifying surface defects and the other to optimizing the process parameters. The first issue concerns the methodology for detecting surface defects and distinguishing between significant and insignificant defects. The second issue impacts the reduction in steel production costs and the improvement in product properties. The modeling of the steel production process is also presented in the work [20]. The paper introduces three models based on process data, which enable the determination of the indicators related to the efficiency of the steel production process. The developed models allow optimization of the process parameters to achieve optimum efficiency and product quality [20].

The fuzzy logic method has been employed to model industrial processes, such as classification, comminution, and combustion in industrial boilers [21,22,23]. The results are promising, which prompted the use of this method in modeling metallurgical processes. Considering the above, this paper presents the application of the fuzzy logic approach as one of the leading AI methods for modeling heating and scale formation processes in an industrial reheating furnace.

## 2. Materials and Methods

Scale is the result of the steel reaction with the gaseous components of the furnace atmosphere. According to the authors of [24,25], scale is the layer of oxidation product formed on the metal surface in the first seconds of the process of the order of 100 nm in thickness. The outer layer of the scale is compact, while the inner layer is porous, generally inhibiting the rate of oxidation [24,26]. At low temperatures, the reaction is completely inhibited, while at high temperatures, the oxidation process continues due to the diffusion of reacting substrates through the product layer. Both thermodynamic considerations [27] and research results [28] indicate that the scale formed during steel heating to the temperature required for forming consists of three iron oxides, which occur in the scale as three parallel layers in the order corresponding to the oxygen content [29,30]:

- FeO (wüstite) with the lowest oxygen content,
- Fe_3_O_4_ (magnetite),
- Fe_2_O_3_ (haematite).

Temperature has a powerful influence on scale formation. The rate of scale formation at temperatures up to about 870 K is very low and insignificant; nevertheless, once this temperature is exceeded, it grows rapidly [12].

Limiting the scale formed is essential in reducing heat consumption in heating steel charges by limiting the scald by selecting an appropriate heating technology [31]. The forming scale layer affects the heat transfer to the charge. Compared to steel, the scale has a much lower thermal conductivity coefficient [32], which worsens the heating intensity, raises heat consumption (while maintaining the target final temperature), or results in lower final temperatures in the cross-section of the charge (with the same amount of supplied heat).

In this study, the test object was a reheating furnace used to heat a steel charge to the appropriate temperature before further processing. In furnaces that heat the charge for hot forming, the following zones are distinguished [33]:

- regeneration zone—a burner-free zone that utilizes the enthalpy of flue gasses,
- preheating zone—slow heating of the charge to a temperature of 850–1000 °C,
- heating zone—rapid heating of the charge surface to a temperature of 1150–1250 °C,
- equalization zone—temperature equalization in the cross-section of the charge.

**Figure 1:**
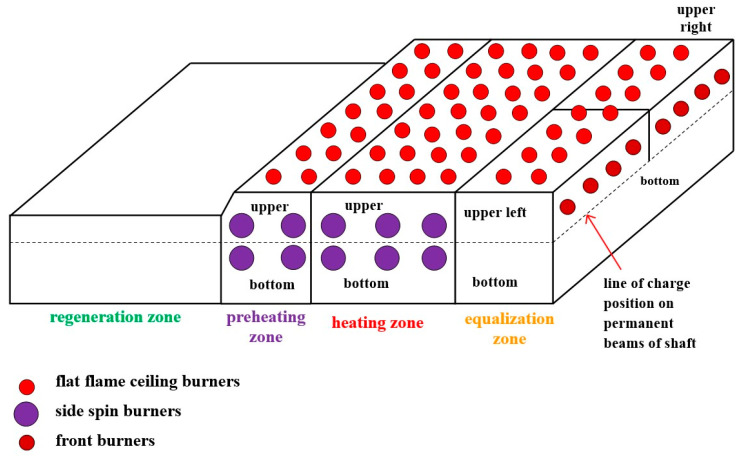
Diagram of an example walking beam furnace.

The research was carried out in walking beam furnaces operating in hot rolling mill departments. Walking beam furnaces are furnaces where the heated charge moves stepwise along the hearth from the charge side to the discharge side using a pusher [34,35,36]. A characteristic feature of walking beam furnaces is the sliding rails along which the charge moves. These furnaces are equipped with flat-flame burners, often also with side burners, and sometimes even with front burners in the equalization zone [33]. The charge in the furnace is placed at intervals, which results, on the one hand, in better heating of the charge and, on the other hand, in reduced unit efficiency of the furnace owing to the lower degree of utilization of the hearth’s heating surface. This paper analyses the results of heating tests of walking beam furnaces with different efficiency levels. A diagram of an example walking beam furnace is shown in Figure 1.

Heating steel charges are essential for the proper course of the subsequent process in the technological sequence—forming the charge. At the same time, it is a highly energy-intensive process. Therefore, various physical parameters related to the process and the charge are adjusted to optimize energy efficiency. These parameters include:

- initial charge temperature *t*_*w*_′, °C,
- heating time *τ*, min,
- excess combustion air ratio value λ,
- initial thickness of the scale layer *δ*_*zg*_′, mm.

These parameters are also inputs in the analyzed process model.

During the study, the initial temperature of the charge (before it was introduced into the furnace) was measured while it was on the loading platform. The measurement results, obtained using a total radiation pyrometer, were taken from the furnace instrumentation and verified with a Thermacam P65 FLIR thermal imaging camera (FLIR SYSTEMS, Warsaw, Poland). In the case of the thermal imaging camera, each of the acquired thermograms was subjected to detailed analysis, providing information on the quantitative temperature distribution on the surface of the charge. Subsequently, the surface temperature of the charge was determined for each case. The measurement of the surface temperature of the charge at the furnace exit was subject to similar procedures, except that the thermograms were recorded while the charge was in motion. Similarly, the surface temperature of the charge at the furnace exit was measured using a pyrometer.

The heating process time depends on the furnace’s efficiency, and its value was determined based on the instrumentation readings. The excess air ratio was determined using MRU flue gas analyzers for natural gas combustion. The initial thickness of the scale layer was determined by taking several samples of scale from the surface of the charge entering the furnace and measuring the thickness with a micrometer. The obtained values were averaged to receive the most reliable result.

**Figure 2:**
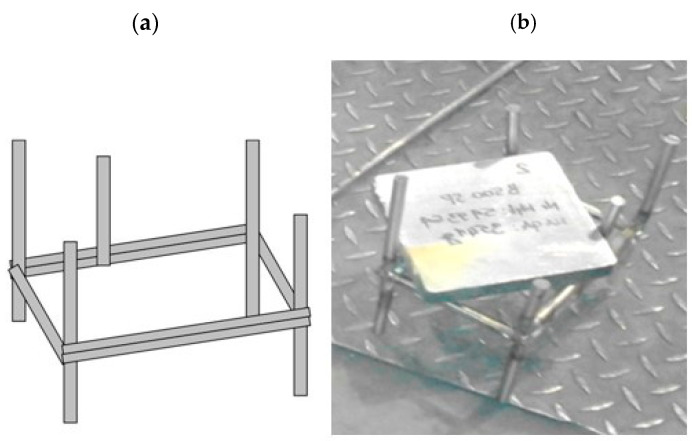
View of special rack (**a**) and rack with a sample (**b**).

A steel sample obtained by cutting from a given cast batch was cooled and then weighed using an Axis B15 scale (AXIS, Gdansk, Poland) (m_0_). This is a technical scale with a maximum load of 15,000 g and a reading accuracy of 1 g. The minimum load of the scale is 100 g. The scale is in accuracy class III, and the weighing time is less than 3 s. The sample was introduced into the furnace in a specially designed basket (Figure 2) and placed on a heated slab. After the entire heating cycle, the sample was cooled, weighed again (m_1_), and then scaled by mechanical cleaning utilizing an orbital sander. After complete scale removal, the sample was weighed again (m_2_). The measurement results were entered into a computerized measurement database and then subjected to a calculation process.

**Figure 3:**
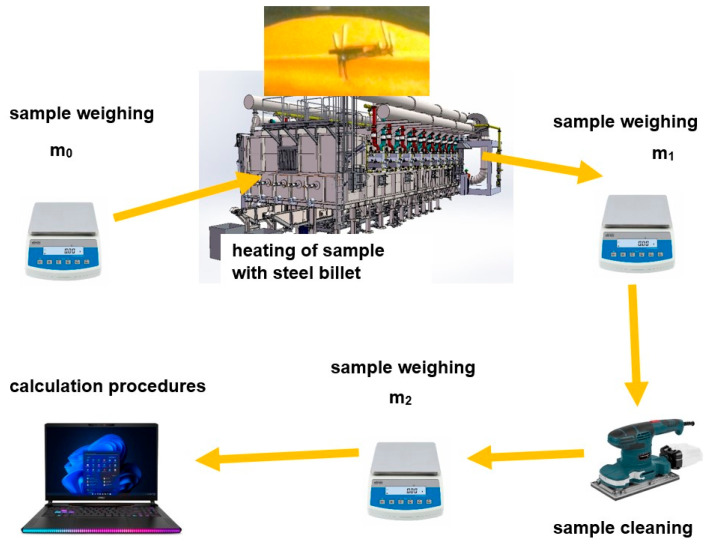
Scheme of methodology for measuring amount of scale.

A diagram of the measurement methodology is shown in Figure 3.

The amount of scale formed during the heating of a single billet can be determined using the following equation [7]:

(1) $$z^{\prime }=\frac{m_{1}-m_{2}}{1000\cdot A},\mathrm{kg}/m^{2}$$

The steel loss due to scale formation is calculated using the equation:

(2) $$z=\frac{m_{0}-m_{2}}{1000\cdot A},\mathrm{kg}/m^{2}$$

The thickness of the scale layer is determined using the following equation [7]:

(3) $$\delta_{zg.}=\frac{z}{\rho_{zg.}\cdot {\bar{X}}_{Fe}},m$$

Thus, knowing the thickness of the scale layer, the steel loss can be determined [7]:

(4) $$z=\delta_{zg}\cdot \rho_{zg}\cdot {\bar{X}}_{Fe},\mathrm{kg}/m^{2}$$

The amount of scale formed during the heating of a single billet can be determined using the equation [7]:

(5) $$z^{\prime }=\frac{z}{{\bar{X}}_{Fe}}\cdot A_{w},\mathrm{kg}$$

(6) $$A_{w}=2\cdot b\cdot h+2\cdot b\cdot l+2\cdot h\cdot l,m^{2}$$

The volume of the charge can be ascertained as follows:

(7) $$V_{w}=b\cdot h\cdot l,m^{3}$$

The mass of the charge will be:

(8) $$m_{w}=V_{w}\cdot \rho_{w},\mathrm{kg}$$

The amount of scale in relation to the charge mass will be [7]:

(9) $$z_{zg}^{\prime \prime }=\frac{z^{\prime }}{m_{w}},{\mathrm{kg}}_{\mathrm{zg}}/{\mathrm{kg}}_{w}$$

The fuzzy logic method for modeling industrial processes was used in optimization studies. This paper introduces a heating and scale formation model to minimize the excess energy consumption for heating a steel charge in an industrial furnace before forming. The model focuses on reducing the scale layer’s thickness and the charge’s final surface temperature.

The fuzzy logic-based approach constitutes an artificial intelligence (AI) method for expressing the sophisticated behavior of complex systems [20,21,22,23,37]. The technique is proficient in managing the uncertainties and imprecisions inherent in complex systems like the one considered in the paper. The developed model considers several input parameters, such as the furnace capacity, charge size, initial surface temperature of the charge, heating time of the charge, combustion excess air ratio, and the initial thickness of the scale layer, which introduce uncertainty and inaccuracy. The model can handle uncertainty and inaccuracy by incorporating linguistic variables and membership functions. In contrast, traditional deterministic models may struggle to accommodate these variabilities, potentially leading to less reliable outcomes.

Fuzzy logic provides an approach that incorporates expert knowledge and handles uncertainties, which is crucial for modeling the sophisticated behavior of the complex systems considered in the paper. The following inputs are considered in the developed HSF model:

1. Furnace capacity, W.
2. Charge size, BS.
3. Initial surface temperature of the charge, T_in_.
4. Heating time of the charge, t, min.
5. Combustion excess air ratio, EA.
6. Initial thickness of the scale layer, S_in_.

The HSF model enables the prediction of two outputs, mainly:

1. Final surface temperature of the charge, T_out_.
2. Final thickness of the scale layer, S_out_.

The inputs and outputs of the model are described in Table 1.

According to Table 1, the comprehensive HSF model allows for considering furnaces with capacities between 140 and 250 t/h. Four different charge sizes, encoded as “1”, “2”, “3” and “4” in the HSF model, with the following dimensions, are used to build the model:

“1”: 0.16 m × 0.16 m × 12 m (charge volume 0.3072 m^3^).

“2”: 0.16 m × 0.16 m × 15 m (charge volume 0.384 m^3^).

“3”: 0.18 m × 0.18 m × 15 m (charge volume 0.486 m^3^).

“4”: 0.28 m × 0.22 m × 15 m (charge volume 0.924 m^3^).

**Figure 4:**
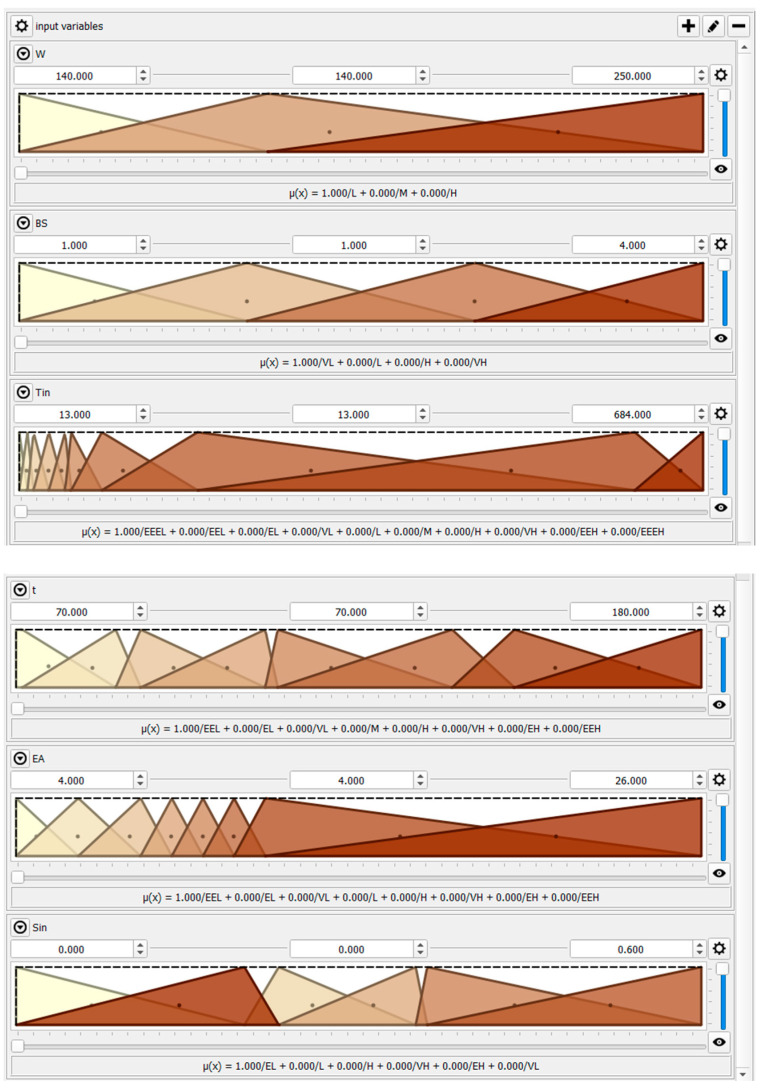
Input parameters for the HSF model.

**Figure 5:**
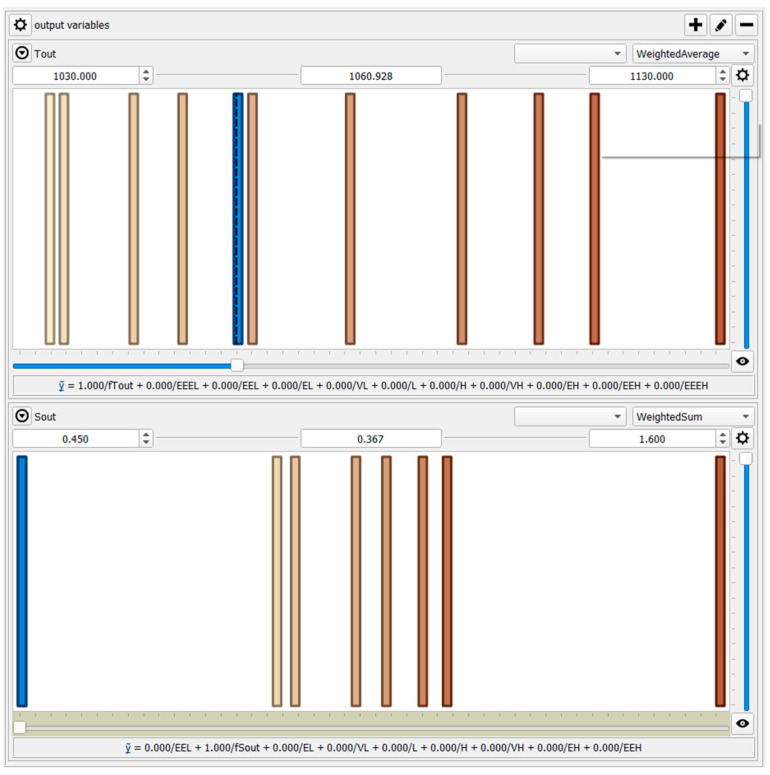
Output parameters for the HSF model.

All the inputs are expressed by overlapping triangular fuzzy sets (Figure 4). At the same time, the outputs are formulated by constant functions (Figure 5), according to the Takagi–Sugeno inference engine employed in the HSF paper [38,39,40,41].

Appendix A provides the membership functions for the input and output parameters. These functions are defined based on empirical data and expert knowledge about the system’s behavior. We used triangular membership functions, following the principles outlined in the work [37,42,43,44,45], due to their simplicity and effectiveness in representing variables with gradual transitions between fuzzy sets. The shape of each membership function is determined through a combination of data analysis and system requirements, covering all the domains of the input parameters. The model’s logic allows us to describe the process’s behavior, so the proper if–then rule base should be implemented [37,38]. The developed HSF model applied the following rule base (Table 2).

The fuzzy rule base contains 14 rules, including all the membership functions and mapping inputs for the output domain. These “IF–THEN” rules combine inputs using logical conjunction by the “AND” operation, allowing the reproduction of specific conditions and behavior of the complex system [37,41]. Besides common easy to interpret fuzzy sets, such as EL, VL, L, M, H, VH, and EH, which stand for extremely low, very low, low, medium, high, very high, and extremely high, respectively, other linguistic terms (EEEL, EEL and EEH, EEEH) are used to express lower and higher values than EL and EH. Finally, two additional functions, i.e., fTout and fSout, are introduced to better describe the model’s behavior in the unknown areas of the input domain [38,39,40,41].

(10) $$fTout=0.956\cdot W-5.147\cdot BS-0.014\cdot T_{in}-0.119\cdot t+0.306EA-103.476\cdot S_{in}+939.523,$$

(11) $$fSout=-0.004\cdot W+0.004\cdot t+0.003EA+2.439\cdot S_{in}+0.635$$

**Figure 6:**
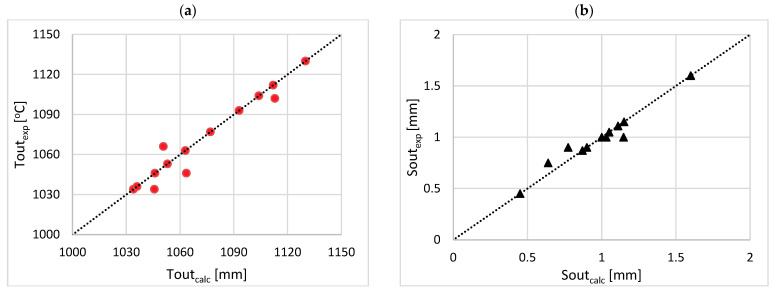
Comparison between measured and calculated results for (**a**) final surface temperature of the charge and (**b**) final thickness of scale layer.

Such a developed model allows for predicting the outputs, i.e., the final surface temperature of the charge (Tout) and the final thickness of the scale layer (Sout). Figure 6 compares the measured and calculated results.

The accuracy of the developed HSF model is acceptable. The maximum relative error between the measured and calculated data is lower than 2% for Tout and 15% for Sout. Thus, the developed HSF model can be used to predict the influence of the operational parameters on the outputs and allows the prediction of both the final surface temperature of the charge and the final thickness of the scale layer.

## 3. Results and Discussion

As the performance optimization of the charge heating process needs to be considered with its energy efficiency, the most significant economic effect will be achieved for the lowest input values, i.e., W = 140 t/h, BS = 1 (0.16 m × 0.16 m × 12 m), Tin = 13 °C, t = 70 min, EA = 4%, Sin = 0 mm. For this set of inputs, the final surface temperature of the charge and the final thickness of the scale layer are equal to Tout = 1061 °C and 0.367 mm. Increasing the furnace capacity from 140 t/h to 180 t/h and 240 t/h will enhance the final surface temperature of the charge as the higher nominal capacity corresponds to the higher furnace temperature (Table 3).

However, considering an initial thickness of the scale layer (S_in_) equal to zero is meaningless, as we can already speak of scale when the oxidation product layer formed on the metal surface is of the order of 100 nm.

Table 4 shows the influence of the furnace capacity on the outputs for an initial thickness of the scale layer equal to 0.35 mm. The final surface temperature shows the same trends as before, i.e., it increases with the furnace capacity. Nonetheless, its magnitude is lower owing to the presence of the scale layer, which has an isolating effect on the surface of the charge, deteriorating the heat transfer conditions.

The scale layer affects the heat transfer to the charge as the scale has a much lower thermal conductivity coefficient than steel [34]. Therefore, the scale worsens the heating intensity, increases heat consumption, and results in lower final temperatures in the cross-section of the charge, which lowers the furnace’s thermal efficiency. This has practical implications in the industry in maintaining optimal heating times of the charge, augmenting the heat transfer intensity, and raising the temperature in the working chambers of the furnaces [46,47]. An interesting effect can be noticed for the final thickness of the scale layer, which decreases with the increase in furnace capacity. Since the diffusion of reactants within the scale plays a decisive role in the oxidation of metals and alloys [24,26], being one of the slowest partial processes determining the overall reaction rate, the decrease in the final thickness of the scale layer can be explained by the larger dimensions of higher capacity furnace zones. The final thickness of the scale layer also depends on the oxidation temperature and time [48].

In addition to the furnace capacity, the charge size will only influence the final temperature of the charge as the thickness of the scale layer will remain unchanged (Table 5).

An increase in charge size results in a decline in the final surface temperature, as the larger charge dimensions, corresponding to a higher charge volume, accumulate more thermal energy.

Based on the model research evaluation, the results can be the following:

- The model confirms that the most significant economic effect will be achieved for the lowest input values, i.e., W = 140 t/h, BS = 1 (0.16 m × 0.16 m × 12 m), T_in_ = 13 °C, t = 70 min, EA = 4%, S_in_ = 0 mm. It infers that the initial phase of the process is as important as the charge conditions. The smaller initial thickness of the scale layer makes the heating process more economical, contributing to the global decarbonization of the metallurgical processes [49].
- An increase in charge size leads to a drop in the final surface temperature because the larger charge dimensions, which correspond to a higher charge volume, accumulate more thermal energy.
- Increasing the furnace capacity from 140 t/h to 180 t/h and 240 t/h enhances the charge’s final surface temperature since a higher nominal capacity corresponds to a higher furnace temperature.
- It was observed that the final thickness of the scale layer decreases with increasing furnace capacity. This can be attributed to the lower diffusion rates of the reactants in the larger dimensions of higher-capacity furnace chambers.

## 4. Conclusions

The paper introduces a fuzzy logic-based heating and scale formation model in an industrial reheating furnace. Scale formation depends on the temperature of the initial charge, heating time, excess air coefficient value, and initial scale thickness. Steel loss can be determined experimentally; however, the methodology for its measurement is challenging owing to the high temperature of the charge and the need to interfere with the technological process. The process of heating a steel charge in modern industrial furnaces presents extremely complex challenges when creating a determined mathematical model. The use of artificial intelligence gave the possibility of modeling heating and scale-forming processes.

The fuzzy logic-based approach was employed to develop the HSF model. Fuzzy logic provides a strategy that incorporates expert knowledge and handles uncertainties, which is crucial for modeling the sophisticated behavior of the complex systems considered in the paper. According to the Takagi–Sugeno inference engine, all the inputs were expressed by overlapping triangular fuzzy sets, while constant functions formulated the outputs. Two additional functions were introduced to better describe the model’s behavior in the input domain’s unknown areas.

The developed HSF model can predict the influence of the operational parameters on the outputs. It allows the prediction of the charge’s final surface temperature and the scale layer’s final thickness, determining energy consumption, and product loss. The model was successfully validated with experimental results from industrial-scale furnaces.

Artificial intelligence minimizes the number of necessary experimental studies while also enabling the construction of a model that reliably describes the analyzed process, is statistically assessable for conformity, and can be further modified.

## Figures and Tables

**Table 1:** Inputs and outputs of the HSF model.

Parameter	Values
Inputs	
Furnace capacity W, t/h	140–250
Charge size BS	1–4
Initial surface temperature of the charge T_in_, °C	13–684
Heating time of the charge t, min	70–180
Combustion air ratio EA	4–26
Initial thickness of the scale layer S_in_, mm	0–0.6
Outputs	
Final surface temperature of the charge T_out_, °C	1030–1130
Final thickness of the scale layer S_out_, mm	0.450–1.6

**Table 2:** Fuzzy rule base of the HSF model.

ID	Rule
1	If W is L and BS is VL and Tin is L and t is H and EA is VL and Sin is EL then Tout is VL and Sout is EEL
2	if W is H and BS is H and Tin is EEH and t is EH and EA is H and Sin is L then Tout is EEEH and Sout is EL
3	if W is M and BS is VH and Tin is EEEH and t is EL and EA is L and Sin is VL then Tout is EL and Sout is VL
4	if W is M and BS is L and Tin is EEEL and t is VL and EA is EL and Sin is H then Tout is EEEL and Sout is L
5	if W is M and BS is L and Tin is EEL and t is VL and EA is EL and Sin is H then Tout is EL and Sout is L
6	if W is M and BS is VH and Tin is EEL and t is VL and EA is EL and Sin is H then Tout is EL and Sout is L
7	if W is M and BS is L and Tin is VL and t is EEL and EA is EEL and Sin is H then Tout is H and Sout is L
8	if W is M and BS is VH and Tin is VL and t is EEL and EA is EEL and Sin is H then Tout is H and Sout is L
9	if W is H and BS is H and Tin is VH and t is VH and EA is EEH and Sin is VH then Tout is EEH and Sout is H
10	if W is H and BS is H and Tin is M and t is EEH and EA is VL and Sin is VH then Tout is EH and Sout is VH
11	if W is M and BS is VH and Tin is EL and t is M and EA is EH and Sin is H then Tout is EEL and Sout is EH
12	if W is M and BS is L and Tin is EL and t is M and EA is EH and Sin is H then Tout is L and Sout is EH
13	if W is H and BS is H and Tin is H and t is EH and EA is VH and Sin is EH then Tout is VH and Sout is EEH
14	if W is any and BS is any and Tin is any and t is any and EA is any and Sin is any then Tout is fTout and Sout is fSout

**Table 3:** Effect of furnace capacity without the initial thickness of scale layer.

Parameter	Values		
Inputs			
Furnace capacity W, t/h	140	180	250
Charge size BS	1		
Initial surface temperature of the charge T_in_, °C	13		
Heating time of the charge t, min	70		
Combustion air ratio EA	4		
Initial thickness of the scale layer S_in_, mm	0		
Outputs			
Final surface temperature of the charge T_out_, °C	1061	1099	1166
Final thickness of the scale layer S_out_, mm	0.367		

**Table 4:** Effect of furnace capacity with initial thickness of scale layer.

Parameter	Values		
Inputs			
Furnace capacity W, t/h	140	180	250
Charge size BS	1		
Initial surface temperature of the charge T_in_, °C	13		
Heating time of the charge t, min	70		
Combustion air ratio EA, %	4		
Initial thickness of the scale layer S_in_, mm	0.35		
Outputs			
Final surface temperature of the charge T_out_, °C	1025	1063	1130
Final thickness of the scale layer S_out_, mm	1.22	1.06	0.78

**Table 5:** Effect of charge size.

Parameter	Values			
Inputs				
Furnace capacity W, t/h	180			
Charge size BS	1	2	3	4
Initial surface temperature of the charge T_in_, °C	13			
Heating time of the charge t, min	70			
Combustion air ratio EA, %	4			
Initial thickness of the scale layer S_in_, mm	0.35			
Outputs				
Final surface temperature of the charge T_out_, °C	1063	1058	1053	1048
Final thickness of the scale layer S_out_, mm	1.06			

## Data Availability

The original contributions presented in this study are included in the article. Further inquiries can be directed to the corresponding author.

## Nomenclature

A	sample area, [m^2^]
Aw	outer surface of charge, [m^2^]
b, h, l	steel charge dimensions, [m]
EA, λ	combustion excess air ratio, [-]
m_0_	sample mass before heating, [g]
m_1_	sample mass after heating, [g]
m_2_	sample mass after complete descaling, [g]
Sin	initial thickness of the scale layer, [mm]
Sout	final thickness of the scale layer, [mm]
t	warm-up time of the charge, [min]
Tin	initial surface temperature of the charge, [°C]
Tout	final surface temperature of the charge, [°C]
W	furnace capacity, [t/h]
$\bar{X}$ _Fe_	average mass fraction of iron in scale, ($\bar{X}$_Fe_ = 0.74),
z’	amount of scale, [kg/m^2^],
z	surface loss of steel to scale, [kg/m^2^]
ρ_zg_.	scale density (3900 kg/m^3^)
Acronyms	
AI	Artificial intelligence
BS	Charge size
HSF	Heating and scale formation model

## Appendix A

InputVariable: W
enabled: true
range: 140.000 250.000
lock-range: false
term: L Ramp 180.000 140.000
term: M Triangle 140.000 180.000 250.000
term: H Ramp 180.000 250.000
InputVariable: BS
enabled: true
range: 1.000 4.000
lock-range: false
term: VL Ramp 2.000 1.000
term: L Triangle 1.000 2.000 3.000
term: H Triangle 2.000 3.000 4.000
term: VH Ramp 3.000 4.000
InputVariable: Tin
enabled: true
range: 13.000 684.000
lock-range: false
term: EEEL Ramp 21.000 13.000
term: EEL Triangle 13.000 21.000 27.000
term: EL Triangle 21.000 27.000 42.000
term: VL Triangle 27.000 42.000 58.000
term: L Triangle 42.000 58.000 64.000
term: M Triangle 58.000 64.000 94.000
term: H Triangle 64.000 94.000 188.000
term: VH Triangle 94.000 188.000 617.000
term: EEH Triangle 188.000 617.000 684.000
term: EEEH Ramp 617.000 684.000
InputVariable: t
enabled: true
range: 70.000 180.000
lock-range: false
term: EEL Ramp 86.000 71.000
term: EL Triangle 71.000 86.000 90.000
term: VL Triangle 86.000 90.000 110.000
term: M Triangle 90.000 110.000 112.000
term: H Triangle 110.000 112.000 140.000
term: VH Triangle 112.000 140.000 150.000
term: EH Triangle 140.000 150.000 180.000
term: EEH Ramp 150.000 180.000
InputVariable: EA
enabled: true
range: 4.000 26.000
lock-range: false
term: EEL Ramp 6.000 4.000
term: EL Triangle 4.000 6.000 8.000
term: VL Triangle 6.000 8.000 9.000
term: L Triangle 8.000 9.000 10.000
term: H Triangle 9.000 10.000 11.000
term: VH Triangle 10.000 11.000 12.000
term: EH Triangle 11.000 12.000 26.000
term: EEH Ramp 12.000 26.000
InputVariable: Sin
enabled: true
range: 0.000 0.600
lock-range: false
term: EL Ramp 0.200 0.000
term: L Triangle 0.200 0.230 0.350
term: H Triangle 0.230 0.350 0.360
term: VH Triangle 0.350 0.360 0.600
term: EH Ramp 0.360 0.600
term: VL Triangle 0.000 0.200 0.230
OutputVariable: Tout
enabled: true
range: 1030.000 1130.000
lock-range: false
aggregation: none
defuzzifier: WeightedAverage Automatic
default: nan
lock-previous: false
term: fTout Linear 0.956 −5.147 −0.014 −0.119 0.306 −103.476 939.523
term: EEEL Constant 1034.000
term: EEL Constant 1036.000
term: EL Constant 1046.000
term: VL Constant 1053.000
term: L Constant 1063.000
term: H Constant 1077.000
term: VH Constant 1093.000
term: EH Constant 1104.000
term: EEH Constant 1112.000
term: EEEH Constant 1130.000
OutputVariable: Sout
enabled: true
range: 0.450 1.600
lock-range: false
aggregation: none
defuzzifier: WeightedSum Automatic
default: nan
lock-previous: false
term: EEL Constant 0.450
term: fSout Linear −0.004 0.000 0.000 0.004 0.003 2.439 0.635
term: EL Constant 0.870
term: VL Constant 0.900
term: L Constant 1.000
term: H Constant 1.050
term: VH Constant 1.110
term: EH Constant 1.150
term: EEH Constant 1.600
